# Induction of the epithelial-mesenchymal transition in the endometrium by chronic endometritis in infertile patients

**DOI:** 10.1371/journal.pone.0249775

**Published:** 2021-04-07

**Authors:** Mitsuaki Ishida, Akie Takebayashi, Fuminori Kimura, Akiko Nakamura, Jun Kitazawa, Aina Morimune, Tetsuro Hanada, Koji Tsuta, Takashi Murakami

**Affiliations:** 1 Department of Pathology and Laboratory Medicine, Kansai Medical University, Osaka, Japan; 2 Department of Obstetrics and Gynecology, Shiga University of Medical Science, Otsu, Shiga, Japan; Fondazione IRCCS Ca’ Granda Ospedale Maggiore Policlinico, ITALY

## Abstract

**Background:**

The purpose of the present study was to evaluate the relationship between chronic endometritis and the epithelial-mesenchymal transition in the endometrium of infertile patients in the implantation phase.

**Methods:**

Endometrial biopsy specimens from 66 infertility patients were analyzed. The presence of chronic endometritis was investigated by immunostaining for CD138. Immunohistochemical staining for E-cadherin, N-cadherin, Slug, and Snail was performed, and the expression profiles were statistically analyzed according to the presence of chronic endometritis. When the loss of E-cadherin expression and/or the positive expression of N-cadherin was detected, the specimen was considered epithelial-mesenchymal transition-positive. Epithelial-mesenchymal transition-positive cases were also statistically analyzed according to the presence of chronic endometritis. The characteristics of the patients in the epithelial-mesenchymal transition-positive and epithelial-mesenchymal transition-negative groups were compared. The association between variables, including age, body mass index, gravidity, parity, and each causative factor of infertility and epithelial-mesenchymal transition positivity was analyzed.

**Results:**

The rates of the loss of E-cadherin expression, the gain of N-cadherin and epithelial-mesenchymal transition positivity were significantly higher in chronic endometritis patients. The expression of Slug, cytoplasmic Snail, and nuclear Snail was also detected at significantly higher rates in chronic endometritis patients. Chronic endometritis were related to the epithelial-mesenchymal transition.

**Conclusion:**

The epithelial-mesenchymal transition was frequently detected in the endometrium in infertile patients with chronic endometritis. Since the epithelial-mesenchymal transition is associated with chronic endometritis, the epithelial-mesenchymal transition appears to be involved in the alteration of mechanisms of implantation.

## Background

Chronic endometritis (CE) is a persistent chronic inflammatory process of the endometrium. CE is usually asymptomatic or presents with only subtle symptoms, including abnormal uterine bleeding, dyspareunia, leucorrhea, and pelvic pain. It has not received much attention, since it was thought that the diagnosis was of little significance [1–3]. However, recent studies have focused on the association between CE and various gynecological conditions, and have shown that CE has a positive relationship with infertility, implantation failure and miscarriage [4–8]. For these reasons, CE is more frequently diagnosed in infertile patients in recent clinical practice. CE is diagnosed pathologically using collected endometrial tissue. It is diagnosed based on pathological findings such as premature decidualization, developmental differences between the gland and stroma, and the infiltration of plasma cells that do not appear in the normal, non-CE, endometrial stromal area [1, 9]. However, at present, there is no global diagnostic standard for CE based on clinical data, such as the implantation rate, and it is mainly diagnosed based on the presence of plasma cells in the endometrial stroma. It has been reported that immunostaining of CD138 (syndecan-1) reveals the presence of plasma cells and is a useful method for the diagnosis of CE [10, 11]. CE is diagnosed in this way based on the presence of plasma cells, which is considered to be a symbol of chronic inflammation. However, the mechanism of impaired implantation related to chronic inflammation has not yet been completely clarified, although the abnormal distribution of immunocompetent cells and the modification of decidualization have been reported in the endometrium with CE [11–14].

The epithelial-mesenchymal transition (EMT) is a process by which polarized epithelial cells lose polarity and intercellular contraction and acquire mesenchymal cell motility [15]. It is well known that the EMT plays crucial roles not only in normal embryological development, but also in several pathological conditions such as wound healing, fibrosis, and cancer development [16–19]. Focusing on the endometrium during the implantation phase, the mesenchymal-epithelial transition (MET), which is the reverse process to the EMT, is induced in the endometrium and promotes the acceptance of embryos to the uterus [20, 21]. The EMT also plays an important role in implantation [22, 23]. On the other hand, the appearance of the EMT is closely related to the presence of inflammation. However, to date, no studies have evaluated the status of the endometrial EMT in the implantation phase in humans, although the identification of microRNA and Progesterone Receptor-regulated genes associated with the EMT in the window of implantation have already been reported [24].

Thus, the occurrence of the EMT with or without CE was analyzed in infertile patients to elucidate the pathophysiology of CE that causes impaired implantation. In addition, the effect of the EMT on infertility was evaluated by examining the cause(s) of infertility that were associated with the occurrence of the EMT in the endometrium.

## Methods

### Ethics

This study conformed to the Clinical Research Guideline of Shiga University of Medical Science and was approved by the research ethics committee (IRB ethics committee approval number, R2014-090). Written, informed consent to participate in this study was obtained from all patients.

### Patients and endometrial sampling

Infertile patients referred to the Department of Obstetrics and Gynecology of Shiga University of Medical Science from April 2015 to March 2018 were enrolled.

Only patients who received *in vitro* fertilization (IVF) were selected for this study. At our institute, hysteroscopy has been performed, in principle, to detect abnormal findings in the uterine cavity, such as morphological abnormalities, submucosal myoma and endometrial polyp before embryo transfer. Endometrial tissue sampling was performed for patients who desired endometrial scratching, and/or an examination for the presence of CE. At the menstrual cycle of the test, the patients were contraceptive.

Patients suffering from endocrine and autoimmune diseases, uterine malformation (e.g., septate uterus), submucous myoma, endometrial polyp detected by hysteroscopy, hydrosalpinx detected by ultrasonography or adenomyosis with over a uterine wall thickness of >2.5 cm at the time that hysteroscopy was performed for the diagnosis of CE were excluded from this study [25–29]. Patients who had received ≥7 days of antibiotics for the treatment of CE were also excluded [4].

The patients underwent a urine LH test provided by our hospital every day for three to four days prior to the estimated ovulation day. On the day that the urine test became positive or the following day, they visited our hospital and the growth status of the follicle was examined. If the growth of the follicle was sufficient as a preovulatory follicle, the date of ovulation was estimated and the patient was scheduled to undergo hysteroscopy and curettage at 7–9 days after ovulation. On the day of endometrial sampling, the patient’s blood was sampled to measure serum levels of estradiol (E_2_) and progesterone (P_4_). Patients with E_2_ <50 pg/mL and P4 <8 ng/mL were excluded. Endometrial tissue obtained by the first curettage from each patient was used in this study. The biopsy specimens were immediately fixed in 10% buffered formalin.

Clinical information, including age, gravidity, parity, body mass index (BMI), serum levels of E_2_ and P_4_, and the cause of infertility were obtained from the clinical records. The diagnosis of male factor, ovarian factor, immune factor and fertilization failure were made by the general methods [30, 31]. The diagnosis of male factor was made as a cause of infertility based on a semen analysis and judged based on the criteria of the WHO Manual, 5th edition [32]. The patient was diagnosed with ovarian factor when the patient suffered from ovulatory dysfunction and premature ovarian insufficiency, as described by Farquhar et al. [33]. When sperm immobilizing antibodies were detected in the patient’s serum, the patient was diagnosed with immune factor as a cause of infertility [34]. Fertilization failure was diagnosed when the failure of fertilization occurred in all oocytes [35]. In the present study, endometriosis was diagnosed when the existence of endometrioma was confirmed by MRI or endometriosis was confirmed and/or treated by laparotomy or laparoscopy within 3 years before participation in this study. In addition, the tubal factor was determined when abnormalities such as obstruction, lift-up and peri-fimbria adhesion were suspected on hysterosalpingography (HSG) and when fallopian tube abnormality was observed on laparoscopy. In the present study, the diagnosis of unexplained infertility was made when the above diagnosis was ruled out [36].

### Immunohistochemistry

Formalin-fixed, paraffin-embedded tissue blocks of the biopsied specimens were cut into 3-μm-thick sections, deparaffinized, and rehydrated. Each section was used for immunostaining. Immunohistochemical analyses were performed using an autostainer (Benchmark XT system; Roche Diagnostics, Basel, Switzerland) according to the manufacturer’s instructions. The primary antibodies used in this study were as follows: mouse monoclonal antibody against CD138 (clone B-A38, 1:100, Cell Marque, CA, USA); mouse monoclonal antibody against E-cadherin (clone 36, pre-diluted, Roche Diagnostics); mouse monoclonal antibody against N-cadherin (clone 6G11, 1:25, DAKO Japan Co. Ltd., Kyoto, Japan); rabbit polyclonal antibody against Slug (ab38551, 1:500, Abcam, Cambridge, UK); and rabbit polyclonal antibody against Snail (ab180714, 1:100, Abcam). I-VIEW DAB universal kit (cat-no. 518100032) was used as a detection system.

Negative controls consisted of slides run with an unspecific mouse IgG (I-2000-1, Vector Laboratories, CA, USA) or unspecific rabbit IgG (I-1000-5, Vector Laboratories).

### Analyses of immunostaining

In this study, diagnosis of CE was performed by pathologist using the immunostaining for CD138. According to previous reports, the number of CD138-positive plasma cells under high-power fields (magnification, ×400) was counted in 10 non-overlapping, random endometrial stromal areas. CE was diagnosed when one or more CD138-positive plasma cells were detected by a pathologist in the endometrial stroma [8, 14].

All fields of each specimen were analyzed for detecting all markers. Membranous immunoreactivity for E- and N-cadherin was evaluated as positive staining. In the present study, when one or more cell clusters were not stained with E-cadherin in the endometrial glands, it was considered a loss of E-cadherin, because normal endometrial glands diffusely expressed E-cadherin. Similarly, the partial expression of N-cadherin was defined as a gain of N-cadherin expression in the present study, because the diffuse expression of N-cadherin was not observed in the present study. We defined a loss of E-cadherin expression or a gain of N-cadherin expression based on presence of the abovementioned changes in more than one gland.

Nuclear immunoreactivity for Slug was evaluated as positive staining, and positive staining in the endometrial glandular cells and stromal cells was analyzed separately. Moreover, nuclear or cytoplasmic immunoreactivity for Snail was evaluated as positive staining, and were analyzed separately.

### Power analysis

We calculated the number of patients required for enrollment using a software program provided by the Department of Biostatistics, Vanderbilt University (http://biostat.mc.vanderbilt.edu/wiki/Main/PowerSampleSize). No studies have statistically compared the incidence of EMT in CE. In addition, since no similar study has yet been published, the number of cases was examined based on the results of studies examining the live birth rates of the cured CE group and the persistent CE group after the treatment of CE. According to the results of this study, the birth rate of IVF after treatment was 61% and 13% [4]. We selected the following settings to determine the appropriate sample size in the “Dichotomous” section: “independent”, “case-control”, “two proportion”, and “Fisher’s exact test”. We selected 0.05 for α and 0.8 for power. We also selected 0.39 (1–0.61) for P0 (the probability of the outcome for a control patient in prospective studies), and 0.87 (1–0.13) for P1 (the probability of the outcome in an experimental subject in prospective studies), as we assumed that those who had an EMT abnormality would not achieve a live birth. When we chose 1 for the ratio of control to experimental subjects for independent prospective studies, the calculation resulted in sample sizes of 19 cases for the control group and 19 cases for the CE group. Thus, the numbers of subjects in the present study were considered adequate.

### Statistical analysis

Statistical analysis was performed using the Graph Pad Prism 5 software program (GraphPad Software Inc., La Jolla, CA). The normality of distribution in each dataset was checked using the Kolmogorov-Smirnov test, and Student’s *t*-test or the non-parametric Mann-Whitney U test was used to test for significance depending on the distribution pattern. The significance of differences in the loss of E-cadherin expression, the expression of N-cadherin, the loss of E-cadherin expression and/or N-cadherin expression, the expression of stromal Slug, the expression of cytoplasmic Snail, the expression of nuclear Snail, and the expression of both stromal Slug and cytoplasmic Snail was compared between the CE-positive group and CE-negative group using a Fisher analysis.

When the loss of E-cadherin expression and/or the gain of N-cadherin expression was detected, the specimen was designated EMT-positive. The patients’ characteristics, including age, gravidity, parity, BMI, E_2_ and P_4_ levels, cause of infertility, and CE were compared between the EMT-positive and EMT-negative groups.

In addition, since this was a retrospective study, a multivariate logistic regression analysis was performed for 8 explanatory variables, including 7 infertility factors (male factor, tubal factor, endometriosis, ovarian factor, fertilization failure, immune factor and unexplained infertility) and CE or EMT-positive with respect to the objective variable EMT-positive. SSPS statistics (version 25) was used for this analysis. Odds ratios and P values were calculated. *P* values of <0.05 were considered to indicate statistical significance in all analyses.

## Results

**Fig 1:**
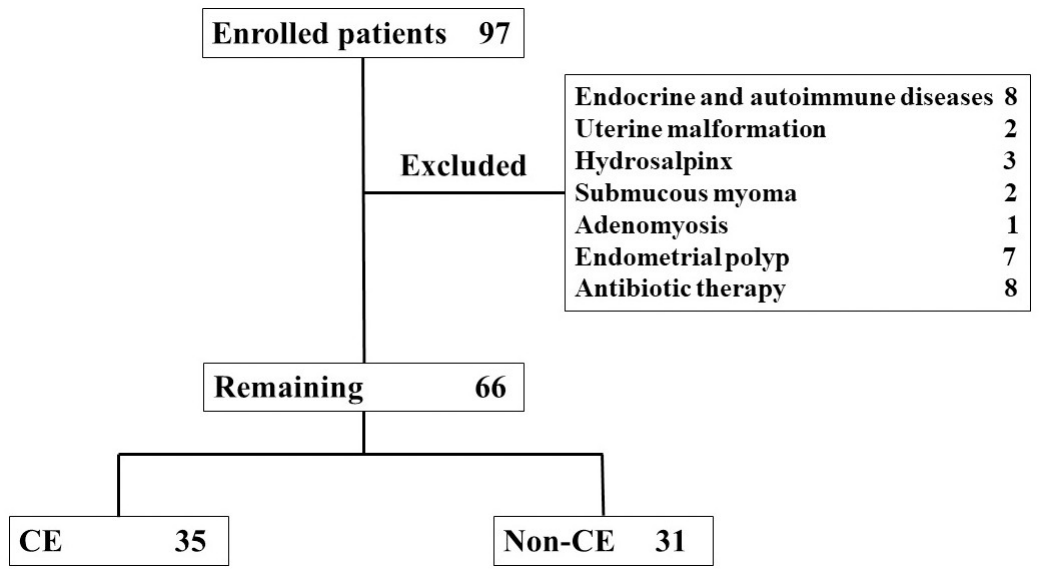
A total of 97 infertility patients who agreed to participate in the study at this time were included in the study. Out of these, 8 patients with endocrine and autoimmune diseases, 2 patients with uterine malformation, 3 patients with hydrosalpinx, 2 patients with submucous myoma, 1 patient with adenomyosis and 7 patients with endometrial polyps were excluded. Eight patients were excluded because of usage of antibiotics. As a result, 31 patients were excluded and 66 patients were included in the present study. CE was identified in 35 of 66 patients.

A total of 97 infertility patients who agreed to participate in the study at this time were included in the study. Out of these, 8 patients with endocrine and autoimmune diseases, 2 patients with uterine malformation, 3 patients with hydrosalpinx, 2 patients with submucous myoma, 1 patient with adenomyosis (the patients also suffered from endometrial polyp) and 7 patients with endometrial polyps were excluded. Eight patients were excluded because of usage of antibiotics (Fig 1). As a result, 31 patients were excluded and 66 patients were included in the present study.

CE was identified in 35 of 66 patients (53.0%). Age, gravidity, parity, BMI, and E_2_ and P_4_ levels did not differ between the CE and Non-CE groups (Table 1). Regarding causes of infertility, among ovarian factor, tubal factor, endometriosis, male factor, fertilization failure, immune factor, and unexplained fertility, the rate of ovarian factor was higher in the Non-CE group (Table 1).

**Table 1:** Patient characteristics in the CE and Non-CE groups.

	CE (35 cases)	Non-CE (31 cases)	*p* value
Age (years)	37.51 ± 0.66	36.39 ± 0.67	0.24
Gravity	0 (0–3)	0 (0–4)	0.97
Parity	0 (0–1)	0 (0–2)	1
Serum level of estradiol	115 (64–356)	111 (63–307)	0.53
Serum level of progesterone	14.7 ± 1.0	15.2 ± 1.2	0.75
Infertility cause			
Ovarian factor	0	5	0.019
Tubal factor	9	10	0.6
Endometriosis	10	6	0.41
Male factor	6	9	0.38
Fertilization failure	3	1	0.62
Immune factor	0	0	1
Unexplained	12	5	0.16

CE, chronic endometritis.

Student’s *t*-test was performed, and the mean and standard error of the mean were shown when the distribution pattern was parametric. The Mann-Whitney U test was used, and the median and minimum-maximum values were shown when the distribution pattern was non-parametric.

### Comparison of the cadherin expression profiles between the CE and Non-CE groups

**Fig 2:**
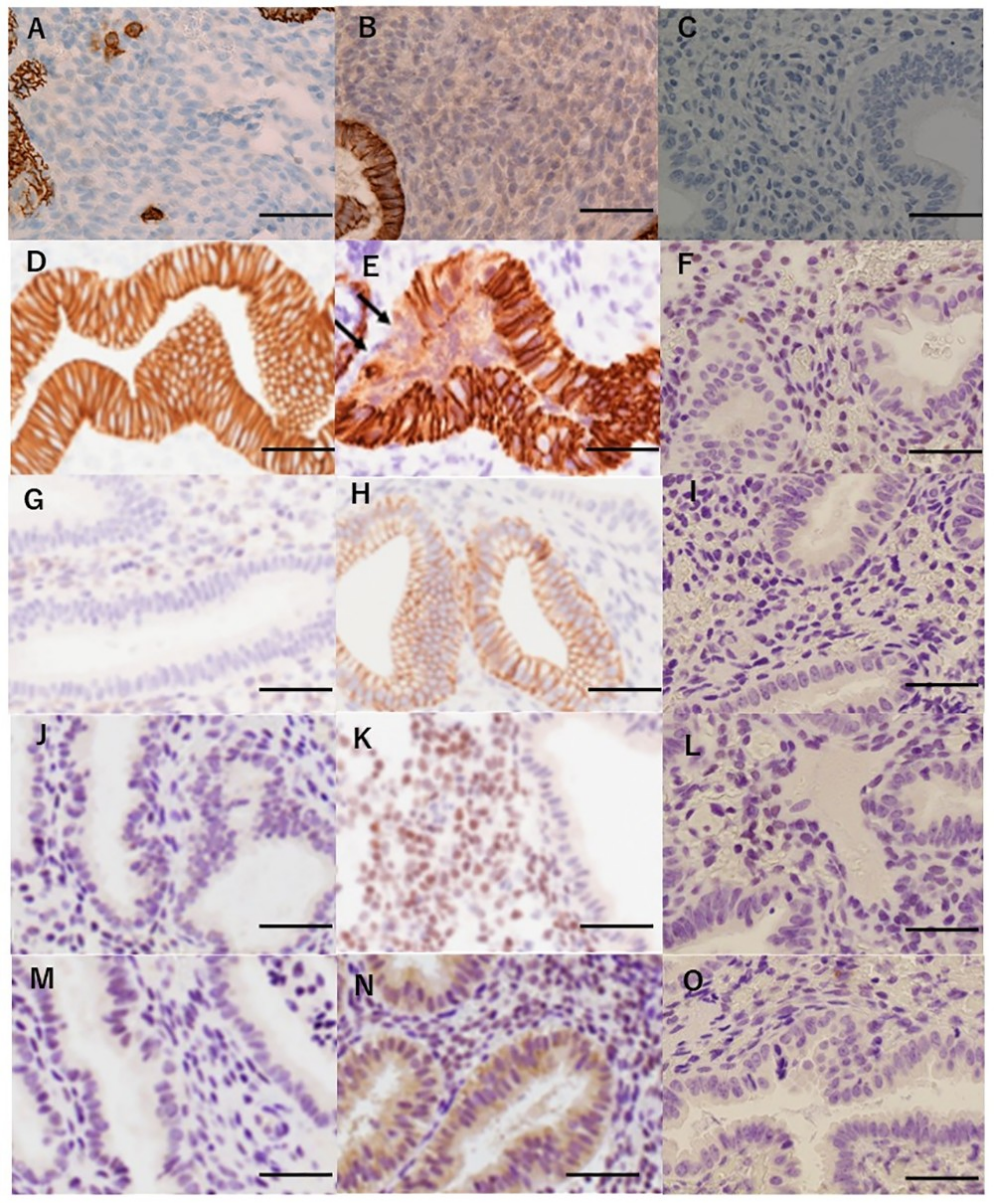
Results of the immunohistochemical analysis of the specimens. (A) Endometrium diagnosed with chronic endometritis. The infiltration of CD138-positive plasma cells was detected in the stromal area of the endometrium by immunohistochemistry. (B) Endometrium diagnosed with non-chronic endometritis. No plasma cells were detected in the stromal area of the endometrium by immunohistochemistry for CD138. (C) Endometrium without CD138 antibody staining. No staining was detected in the endometrium. (D) Endometrium without a loss of E-cadherin. The expression of E-cadherin was detected in all the glandular cells in endometrial specimens. (E) Endometrium with a loss of E-cadherin. A cluster of endometrial glandular cells without E-cadherin staining was observed (arrows). When such a finding was detected, it was judged as a loss of E-cadherin. (F) Endometrium stained without E-cadherin antibodies. No staining was detected in the endometrium. (G) Endometrium without N-cadherin expression. The expression of N-cadherin was not detected in the glandular cells in endometrial specimens. (H) Endometrium with N-cadherin expression. Gain of N-cadherin staining was present in part of the endometrial glandular cells. When positive staining was observed, it was judged as a gain of N-cadherin expression. (I) Endometrium stained without N-cadherin antibodies. No staining was detected in the endometrium. (J) Endometrium without the expression of Slug. Slug was not expressed in the stromal cells of the endometrium. (K) Endometrium with the expression of Slug. Slug was expressed in the nuclei of endometrial stromal cells. When positive staining was observed, it was judged as Slug-positive. (L) Endometrium stained without Slug antibodies detected in the endometrium. (M) Endometrium without expression of Snail. The expression of Snail was not noted in the glandular cells of the endometrium. (N) Endometrium with the expression of Snail. The cytoplasmic expression of Snail was noted in the glandular cells of the endometrium. When positive staining was observed, it was judged as Snail-positive. (O) Endometrium stained without Slug antibodies. No staining was detected in the endometrium. Scale bar represents 50 μm.

Table 2 summarizes the results of the immunohistochemical analyses in the CE and Non-CE groups. The loss of E-cadherin expression in the endometrial glands was observed in 12 of 35 and 2 of 31 cases in the CE and Non-CE groups, respectively (p = 0.0067) (Fig 2A and 2B). N-cadherin-positive glandular cells were seen in 19 and 7 cases in the CE and Non-CE groups, respectively (p = 0.012) (Fig 2C and 2D). The loss of E-cadherin expression and/or N-cadherin expression was observed in 23 and 8 cases in the CE and Non-CE groups, respectively (p = 0.0015). There were no differences in any of the staining patterns between the CE-positive and CE-negative groups when staining was observed.

**Table 2:** The association between chronic endometritis and the cadherin expression profiles in the specimens.

	CE (35 cases)	Non-CE (31 cases)	*p* value
Loss of E-cadherin	12	2	0.0068
Gain of N-cadherin	19	7	0.012
Loss of E-cadherin and/or gain of N-cadherin	23	8	0.0015

CE, chronic endometritis.

A Fisher analysis was used to compare the difference.

### Comparison of the EMT marker expression between the CE and Non-CE groups

Table 3 summarizes the associations between CE and the expression profiles of Slug and Snail. The expression of Slug in stromal cells was seen in 34 of 35 and 21 of 31 cases in the CE and Non-CE groups, respectively (p = 0.0019) (Fig 2E and 2F). No cases showing the expression of Slug in endometrial glandular cells were present in either group. The cytoplasmic expression of Snail was noted in 28 and 11 cases in the CE and Non-CE groups, respectively (p = 0.004) (Fig 2G and 2H), and the nuclear expression of Snail was seen in 6 and 0 cases, respectively (p = 0.026). The expression of both stromal Slug and cytoplasmic Snail was seen in 27 and 8 cases in the CE and Non-CE groups, respectively (p<0.0001). There were no differences in any of the staining patterns between the CE-positive and CE-negative groups when staining was observed.

**Table 3:** The associations between chronic endometritis and the expression of EMT markers in specimens.

	CE (35 cases)	Non-CE (31 cases)	*p* value
Stromal Slug expression	34	21	0.0019
Cytoplasmic Snail expression	28	11	0.0004
Nuclear Snail expression	6	0	0.026
Both stromal Slug + Cyto Snail expression	27	8	<0.0001

CE, chronic endometritis; EMT, epithelial-mesenchymal transition.

A Fisher analysis was used to compare the difference.

### The analysis of variables associated with EMT positivity

Table 4 summarizes the characteristics of the patients in the EMT-positive and EMT-negative groups. Gravidity, parity, BMI, E_2_ and P_4_ levels did not differ between the EMT-positive and EMT-negative groups; however, age tended to be higher in the EMT-positive group. Regarding causes of infertility, among ovarian factor, tubal factor, endometriosis, male factor, fertilization failure, anti-sperm antibody, unexplained fertility, and CE, only CE was higher in the EMT-positive group; the other factors did not differ between the two groups.

**Table 4:** Patient characteristics in the EMT-positive and EMT-negative groups.

	EMT+ (31cases)	EMT- (35 cases)	*p* value
Age (years)	39 (28–44)	37 (27–43)	0.05
Gravity	0 (0–3)	0 (0–4)	0.63
Parity	0 (0–1)	0 (0–2)	0.91
Serum level of estradiol	114 (64–307)	118 (63–356)	0.7
Serum level of progesterone	14.9 ± 1.1	14.95 ± 1.1	0.99
Infertility cause			
Ovarian factor	1	4	0.36
Tubal factor	6	13	0.17
Endometriosis	10	6	0.25
Male factor	5	10	0.26
Fertilization failure	1	3	0.62
Anti-sperm antibody	0	0	1
Unexplained fertility	11	6	0.1
Chronic endometritis	23	12	0.0015

EMT: epithelial-mesenchymal transition.

Student’s *t*-test was performed, and the mean and standard error of the mean were shown when the distribution pattern was parametric. The Mann-Whitney U test was used, and the median and minimum-maximum values were shown when the distribution pattern was non-parametric.

Among the causes of infertility, CE was the only variable associated with EMT positivity in the multivariate logistic analysis (p = 0.005, odds ratio 6.1, 95% CI 1.73–21.6).

## Discussion

In the present study, according to previous reports, the number of CD138-positive plasma cells under high-power fields (magnification, ×400) was counted in 10 non-overlapping, random endometrial stromal areas. CE was diagnosed when one or more CD138-positive plasma cells were detected by a pathologist in the endometrial stroma. The effect of CE on the endometrial EMT in infertile patients was investigated according to this definition, and it was found that the EMT occurred frequently in CE patients, and EMT-related transcription factor was also frequently induced in CE patients.

Among the infertile cause of the patients, the risk factors for EMT were also investigated, and it was found that CE was associated with an increased risk of the induction of the EMT. Thus, CE was the only variable that was closely associated with the induction of the EMT in the implantation-phase endometrium of infertile patients. To the best of our knowledge, this is the first report on the impact of CE on the occurrence of the EMT in the endometrium.

One of the key functions of the endometrium is for embryos to implant and be nourished for the establishment and maintenance of pregnancy. The process of embryo implantation has long been classified into three phases: apposition, attachment, and penetration [37–39]. Apposition is defined as unstable adhesion of the blastocyst to the endometrial surface, when blastocysts and the endometrial apical surface face each other. During this stage, the trophoblasts come close to the luminal epithelium. Then, trophoblasts begin to invade the endometrial surface, in what is called the attachment phase. At this stage, the embryo and endometrium begin cross-talk. A local increase in stromal vascular permeability at the blastocyst attachment site occurs, and rapid morphological changes in the endometrium are initiated. Penetration is a phase that involves the invasion of the embryo into the stromal area through the luminal epithelium to establish a closer relationship with the mother. It has been reported that the EMT and MET are involved at the time of this attachment or penetration [20, 22]. The transformation from EMT to MET does not occur in the normal endometrium; however, it is often seen in other organs [40]. MET is thought to play a central role in the regeneration and decidualization of the endometrium. However, EMT is only involved in the stage of embryo implantation in the normal endometrium when stimulation from the embryo exists. If the EMT occurs in the normal endometrium without an embryo at this stage, it is considered to be some kind of irritation [41, 42].

This study examined the prevalence of the EMT was compared between the CE and non-CE groups. As a result, CE might affect the increased EMT in the implantation-phase endometrium. This is the first article to show the frequent occurrence of EMT in the endometrium of CE patients. This is therefore considered to be the strength of the present article.

On the other hand, these were limitations of the present study with respect to target, the period of sample collection, method, and research items. 1) We did not perform laparoscopy for all patients. Thus, endometriosis was not considered to be a cause of infertility in some of the patients with early-stage endometriosis. In addition, a large number of patients with implantation failure were potentially included in the patients treated with IVF [43, 44]. Since it has been reported that CE is related to implantation failure [45, 46], and because we hypothesized that the occurrence of EMT was related to implantation failure, IVF patients were targeted in the present study. However, the occurrence of the EMT may differ from that in patients who are not infertile. 2) This study examined the effect of CE on the non-physiological EMT in the endometrium by limiting the phase of implantation. We did not examine the occurrence of the EMT in other phases of the menstrual cycle, although this was because the endometrial biopsy specimens could not be unnecessarily obtained from the patient multiple times. 3) The presence of the EMT was determined by immunohistochemistry using tissue samples obtained by endometrial biopsy. Recently, there have been reports that score the status of the EMT according to RNA expression levels and/or next-generation sequencing [47–49]. Moreover, the presence of EMT was not scored; rather, the EMT was only evaluated by its presence or absence in a tissue specimen [50]. 4) We focused on cadherin switch and the transcription factors involved in it, which is considered one of the most important steps of epithelial to mesenchymal transition. We did not study the loss of epithelial markers such as cytokeratin.

The EMT is induced via various signaling cascades, including the transforming growth factor-beta (TGF-β) pathway, a wide range of intracellular signaling pathways induced by TNF-α, the Notch pathway, the Hedgehog pathway and the Wingless and INT-1 signaling pathway [51, 52]. As CE represents a state of persistent inflammation, IL6, IL1β and TNFα are increased during menstrual bleeding [53], and IL1βand TNFα is strongly expressed in the endometrial tissue [54]. On the other hand, TGFβ1, which is associated with immune tolerance, is increased in CE patients with moderate and severe intrauterine adhesion [55]. Both TNFα and TGFβ have been shown to induce transcription factors such as Snail and Twist in immune cells and cancer cells [56–59]. We hypothesize that these are representative mechanisms related to the induction of the EMT in CE and that the EMT could be suppressed by the modulation of these pathways. Furthermore, the EMT might be inhibited indirectly by suppressing inflammation with immunosuppressants [60] and/or directly by EMT inhibitors, such as tranilast [61, 62], which might improve clinical problems such as implantation failure and fibrosis related to CE. We consider these to be subjects for future research.

Moreover, endometrial cancer and endometriosis, as well as CE, are known to be frequent in infertile patients [4, 5, 9, 63–67]. Considering that CE is frequently found in infertile patients, CE—by triggering the EMT—might be the cause of the increased prevalence of endometrial cancer and endometriosis in infertile patients. The EMT is thought to be a central process of cancer invasion and metastasis, and some recent studies have demonstrated an association between endometriosis and the EMT [50, 68–70]. Moreover, regardless of the factors that induce the EMT in stem cells (or non-stem cells), genetic or epigenetic changes caused by endogenous or external stimuli establish a partial EMT and make the hybrid (epithelial-mesenchymal) phenotype stable [71, 72]. Whether the EMT or its reverse process, the MET, enables cells to obtain pluripotency still remains controversial [73], it is necessary to study whether CE affects the development of endometrial cancer or endometriosis through the EMT in the future.

## Conclusion

This is the first report to demonstrate the association between CE and the endometrial EMT. The infertility caused by CE may be due to the high frequency of the EMT in patients with CE and its effect on the endometrial function during implantation.

## Supporting information

S1 Data (XLSX)

## Abbreviations

BMI: body mass index
CE: Chronic endometritis
E_2_: estradiol
EMT: epithelial-mesenchymal transition
MET: mesenchymal-epithelial transition
P_4_: progesterone
TWIST2: twist family basic helix-loop-helix transcription factor 2
